# HIMSS10 – Perspectives from a newcomer pathologist and a seasoned attendee pathologist: Pathologists should attend!

**DOI:** 10.4103/2153-3539.65340

**Published:** 2010-07-13

**Authors:** Alexis B. Carter, Raymond Aller

**Affiliations:** 1 Department of Pathology and Laboratory Medicine, Emory University, 1364 Clifton Road, NE Room, F149A, Atlanta GA, USA; 2 Los Angeles County Department of Public Health and USC School of Medicine, Los Angeles CA, USA

The annual meeting of the Healthcare Information and Management Systems Society (HIMSS – http://www.himss.org ) took place from February 28 to March 4, 2010 in Atlanta, GA, USA. As the largest healthcare information technology conference in the USA, HIMSS10 brought together health IT professionals, clinicians and exhibiting companies who are striving to improve the delivery of patient care with health information technology.[1] While much of the focus of this meeting was directed toward a diversity of clinicians, administrators, vendors and consultants (i.e. non-pathologists and non-laboratorians), there were a number of activities to keep any pathologist informaticist busy throughout the conference.

From the perspective of a newcomer, the first thing to hit, or rather bludgeon, a person while attending this conference was its size. This year 27,855 participants registered with over 900 exhibiting vendors. [1] The vendor area covered one and a half large exhibit halls at the Georgia World Congress Center. It took over 6 hours for one fast-walking pathologist to go through the exhibits, and for the other pathologist, it took 3 days. While much of the vendor area was concentrated on Electronic Health Records (EHRs) for both large and small enterprises, there were a number of laboratory-related and laboratory-focused vendors to peruse. Waterproof keyboards and mice, barcoding systems, laboratory consulting and automated scripting for facilitating testing of healthcare-related software or hardware upgrades were all in attendance.

The keynote sessions had something for anyone involved in healthcare information technology. The newcomer attended the session where Sanjay Gupta, MD (Chief Medical Correspondent, CNN) interviewed Harry Markopolos (the fraud investigator who revealed the Bernard Madoff scandal) regarding his work on fraud in the healthcare setting, particularly with regard to healthcare information technology. He described the process behind federal investigations for fraud as well as some of the methods used to uncover information.

Both of us heard the speech given by Captain Chesley B. “Sully” Sullenberger, III, the captain of US Airways Flight 1549, who landed his crippled passenger jet on the Hudson River and saved the lives of all 155 people on board. He spoke about how the use of the airline industry′s quality and safety practices helped him on that fateful day in January 2009. After having been an airline pilot for over 40 years, he described an era previous to the current quality practices in which all of the airplane staff on board had to remember each individual captain′s preferences rather than a set of streamlined standards. Subordinates reporting an error that a captain had made were simply not done. Since that time, the airline industry has made wide sweeping changes that have been in place for several decades, making them pioneers with regard to quality and safety. Drawing parallels between the previous era and current medical practice, he encouraged medicine to continue to make changes that would allow for a fair and just culture for reporting and addressing errors.

David Blumenthal, MD, MPP, National Coordinator for Health Information Technology with the Department of Health and Human Services also gave a keynote address and attended an Association of Medical Directors of Information Systems (AMDIS) session consisting primarily of Chief Medical Information Officers (CMIOs) to receive input and to address concerns regarding the enacted and proposed legislation stemming from the Health Information Technology for Economic and Clinical Health (HITECH) component of the American Recovery and Reinvestment Act (ARRA).[2,3] 

While the plenary speakers were well known, much of the practical knowledge relevant to our daily practice of pathology informatics was presented at over 350 educational sessions ranging from quality assurance, implementation strategies for new information systems, result communication, staff management, networking technologies, billing, public health and dozens of other topics. Presentations are available free online (http://www.himssconference.org/handouts/) while synchronized audio-slide presentations may be purchased online (http://www.prolibraries.com/himss/). The technical exhibits presented a fascinating glimpse into new developments and maturing technologies, the most significant of which are often to be found in the smallest (10×10) booths, tucked in among the huge companies with which most of us are familiar [Table 1]. The usual number of companies presented an “information-less” booth display (“solving all your connectivity problems”, “meaningful use” and “rapid return on investment”) – can we get that with mayonnaise?

**Table 1:** Key types of technology exhibited at HIMSS10 (the vendors listed are not complete lists, but only illustrative examples)

Biometrics (fingerprint, iris, finger vein, palm vein, voice, others) Automated testing tools Consulting services that focus on “fixing” your less-than-useful system – these typically focus their expertise on working with one popular vendor's system Interface engines – not only were widely known and installed general-purpose tools represented, but also almost every other booth was marketing some type of “integration engine” Alerting systems Electronic medical record systems Health terminology systems “Health information exchange” – everybody claimed to have this Infection-resistant keyboards A few lab-specific vendors (Sunquest, Soft, Psyche, iSoft, 4Medica) Some newly re-emerging LIS vendors – EPIC, Keane, Cattails (Marshfield)

Note: The big Health Information System (HIS) vendors (Mckesson, GE, Eclipsys) did not bring anyone who knew their own laboratory tools. Some of the big HIS folks did not appear to be present (Cerner, Meditech, Siemens)

The experienced attendee of our pair joined HIMSS in 1986 and has attended many of the annual conferences since that time. This meeting continues to be a major benefit to the practice of pathology informatics and for current work in public health informatics. One of the most rewarding aspects is running into familiar faces and getting updates on what is happening in their world. The most prominent expert in the nation on veterinary informatics has moved from University of California at Davis to the National Library of Medicine. Quest Diagnostics has laid off experienced (and well known) people from their informatics group. The CMIO of Kaiser N. California presented an outstanding talk on password security (and why requiring passwords to be changed every 3 months is a *really* bad idea) ... and on ... and on!

In its early years, the proportion of laboratorians at HIMSS was much larger, albeit in a smaller meeting. Of course, at that time most clinicians who asked about their electronic medical record system would describe in glowing terms the laboratory information system. More recently, the presence of pathologists and laboratorians at this meeting was relatively sparse. Organizations such as HIMSS, AMDIS, Association for Pathology Informatics (API), American Medical Informatics Association (AMIA), HL7 (Health Level 7) and IHE (Integrating the Healthcare Enterprise) are important players in developing national policy with regard to healthcare information technology, and input from pathologists and laboratorians is critical to the safety and efficacy of patient care. We encourage any pathologist or other laboratorian who has not recently attended the HIMSS meeting to consider joining the organization and to attend the annual conference next year, which is to be held in Orlando on February 20–24, 2011 (http://www.himssconference.org). Not only can one gain access to a wealth of practical knowledge, but a person can also meet colleagues who will be lifelong partners in enhancing patient care.
